# Prediction of Mechanical Properties of Rare-Earth Magnesium Alloys Based on Convolutional Neural Networks

**DOI:** 10.3390/ma17204956

**Published:** 2024-10-10

**Authors:** Mei Cheng, Xiya Jia, Zhimin Zhang

**Affiliations:** 1 School of Material Science and Engineering, North University of China, 3 Xueyuan Road, Taiyuan 030051, China; 2 Engineering Research Center, Ministry of Education on Magnesium Base Material Processing Technology, 3 Xueyuan Road, Taiyuan 030051, China

**Keywords:** rare-earth magnesium alloys, mechanical properties, microstructure, convolutional neural network

## Abstract

Rare-earth magnesium alloys exhibit higher comprehensive mechanical properties compared to other series of magnesium alloys, effectively expanding their applications in aerospace, weapons, and other fields. In this work, the tensile strength, yield strength, and elongation of a Mg-Gd-Y-Zn-Zr rare-earth magnesium alloy under different process conditions were determined, and a large number of microstructure observations and analyses were carried out for the tensile specimens; a prediction model of the corresponding mechanical properties was established by using a convolutional neural network (CNN), in which the metallographic diagram of the rare-earth magnesium alloy was taken as the input, and the corresponding tensile strength, yield strength, elongation, and three mechanical properties were taken as the output. The stochastic gradient descent (SGD) algorithm was used for parameter optimization and experimental validation, and the results showed that the average relative errors of the tensile strength and yield strength prediction results were 1.90% and 3.14%, respectively, which were smaller than the expected error of 5%.

## 1. Introduction

Magnesium alloys have the advantages of light weight, high specific strength, good electromagnetic shielding performance, and good mechanical processing performance. They are lightweight structural materials with broad application prospects in the fields of automobiles, communications, aerospace, etc. However, their limited strength and plasticity restrict their use in other fields. Therefore, improving the mechanical properties of magnesium alloys is an urgent problem to be solved. In order to improve the efficiency of traditional experimental research, develop new research methods, and accelerate the progress of magnesium alloy research, the use of multidisciplinary cross-integration, computer-aided design and the introduction of artificial intelligence technology have become a new trend in magnesium alloy research.

At present, simulation and optimization methods using finite element analysis have been widely used in material forming. However, due to the highly nonlinear characteristics of the composition, microstructure, processing technology, and mechanical properties of metal materials, it is difficult for traditional prediction models to accurately describe the complex relationship between them [1]. Machine learning (ML) optimizes the performance of computer programs through data or past experience. It has the advantages of a strong learning ability, strong nonlinear approximation ability, and good scalability. Artificial neural networks (ANNs) have been widely used in the field of materials science as a key technology for establishing prediction models for nonlinear systems, especially in the fields of material design [2,3,4], crystal structure [5,6,7], material property correlation analysis [8,9,10], high-throughput material screening [11], material damage and fracture prediction [12,13], and surface roughness [14,15,16]. For example, Zhu et al. [10] constructed a neural network mechanical prediction model to quantitatively describe the relationship between the chemical composition and heat treatment process of low-alloy steel and its mechanical properties. Zhang et al. [8] studied the relationship between the texture and mechanical properties of a magnesium alloy based on an artificial neural network.

There are many factors that affect the mechanical properties of metal materials, among which chemical composition and constituent phases are decisive factors, while processing technology can change the amount and distribution of constituent phases, thereby changing their mechanical properties. Rare-earth magnesium alloys have many main chemical components (at least four or more). In addition to the matrix structure, alloy phase composition also includes compound phases such as Mg_5_Gd and Mg_24_Y_5_ and long-range ordered phases. Therefore, its deformation mechanism is relatively complex. The hcp matrix structure makes the hot deformation of magnesium alloys proceed by twinning and slip processes. During the deformation process, under the obstruction of the second term and grain boundaries, substructures appear inside, causing dynamic recrystallization, which refines the grains and eliminates the deformation texture. Therefore, deformation strengthening is an important strengthening method for magnesium alloys. Studies have shown that hot deformation plus solid solution strengthening can increase the tensile strength of rare-earth magnesium alloys from 200 to 300 MPa in the cast state to more than 400 MPa. Mg-Gd-Y-Zn-Zr rare-earth magnesium alloys were used as the research object, and the experimental data obtained through a series of tensile tests and microstructure observations were used to construct and train the model. According to the prediction results of the artificial neural network, the correlation between microstructure characteristics and tensile properties was analyzed.

## 2. Experimental Materials and Methods

**Figure 1:**
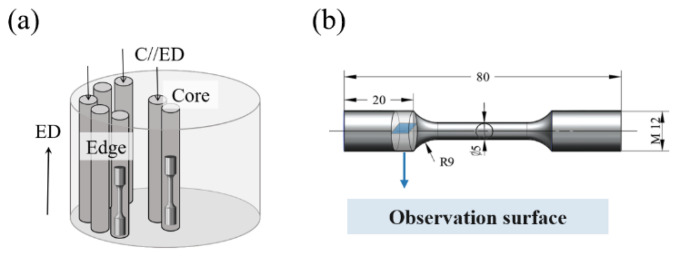
(**a**) Sampling position of tensile rod; (**b**) sample size.

This paper mainly studies the influence of thermal deformation and the microstructure evolution of a rare-earth magnesium alloy on mechanical properties during heat treatment. The samples were cast bars provided by the Shanxi Yinguang Huasheng Magnesium Industry. The initial billet size was a φ330 × 100 mm magnesium alloy bar, and its chemical composition is shown in Table 1. In order to eliminate the inhomogeneity of mechanical properties caused by segregation, magnesium alloy samples after homogenization treatment were tested. First, the cast bars were homogenized at 520 °C × 12 h to eliminate intracrystalline segregation in the cast bars. Then, after three isothermal reciprocating upsetting–extrusion treatments, core and edge samples were taken, respectively, and some samples were annealed at 420 °C × 5 h. As shown in Figure 1, the core, edge, and annealed samples are marked as samples ①, ②, and ③, respectively.

The samples were made into tensile specimens and their shape and size are shown in Figure 1. A tensile test of magnesium alloy specimens was carried out at room temperature using a tensile testing machine (Instron 3382, UK) with a tensile rate of 3 mm/min. The ultimate tensile strength, yield strength, and elongation at break were measured.

## 3. Construction of Neural Network

### 3.1. Overview of Convolutional Neural Network

An artificial neural network is a representative machine learning model generally composed of an input layer, hidden layer and output layer. Each layer can contain multiple neurons. The layers are fully connected. The nonlinear data transmission between layers is completed by weights, biases, and activation functions [17]. However, for images involving a large number of pixel values, it is usually impossible to directly use all pixel values in the fully connected layer, because this will lead to overfitting and increased complexity and difficulty in model convergence. Therefore, a convolutional layer is applied to reduce the dimensionality of image data by finding image features.

**Figure 2:**
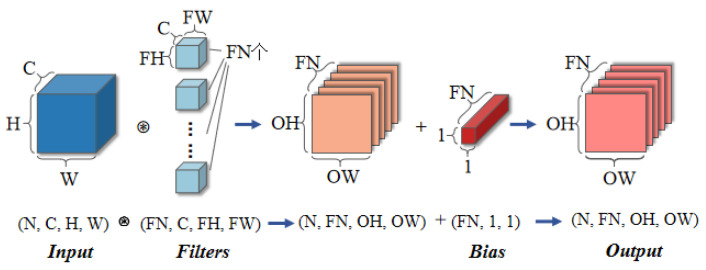
Convolutional operation processing flow.

A convolutional neural network (ConvNet, CNN) is a feedforward neural network with a convolutional structure, which performs well in image segmentation, classification, detection, and retrieval-related tasks [18]. Like other deep neural networks, CNNs consist of an input layer, output layer, and hidden layer. But the main difference lies in the use of hidden layers, which are composed of convolutional layers, pooling layers, and fully connected layers that follow each other. As shown in Figure 2, the convolutional layer extracts local features of the input feature map through filters, reduces the amount of memory occupied by the deep network, effectively reduces the number of network parameters, and alleviates the overfitting problem of the model [19]. The generated map continues to add nonlinear attributes through activation functions. The pooling layer reduces the size of the feature map through pooling operations, such as the maximum value or average value. Finally, a fully connected layer is added to train the features extracted from the image.

To avoid the inability to continue convolution operations after each convolution operation due to space shrinkage, the output size is adjusted by padding and stepping, and the width and height of the feature map after convolution can be expressed as

(1) $$w_{out}=\left(w+2\times PaddingSize-f\right)/s+1\;h_{out}=\left(h+2\times PaddingSize-f\right)/s+1$$

where f denotes the convolution kernel size, h and w denote the height and width of the feature map, respectively, s denotes the step size (stride), and PaddingSize is the padding value.

### 3.2. Data Processing

**Figure 3:**
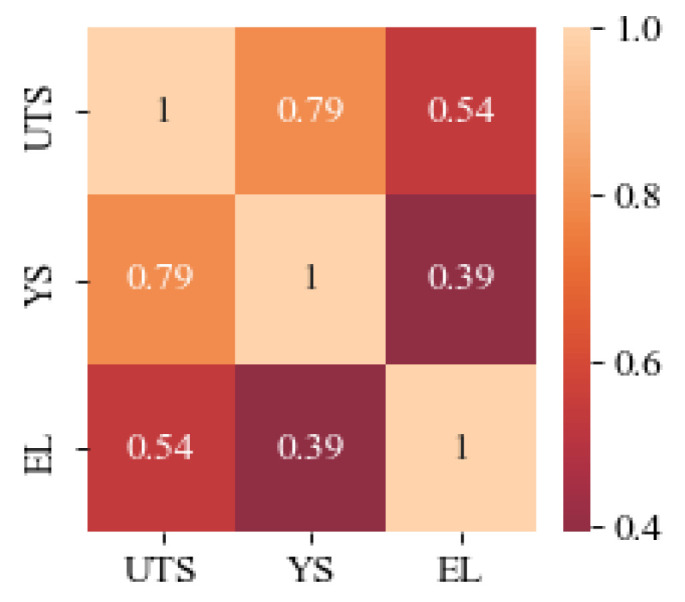
Correlation plot of mechanical properties in the data samples of deformed magnesium alloys.

Current data sources are mainly obtained from two areas: one part comes from reference materials such as domestic and foreign published literature, journals, conference proceedings, etc., and the other part comes from real experimental research. The data of this study come from part of the experimental data of the Center for Precision Forming in the National Defense Science and Technology Industry of North Central University, using an optical microscope (Zeiss Axio Image A2M, DE)with a magnification setting of 500. In total, 200 sets of data on metallographic diagrams and the corresponding mechanical properties of deformed magnesium alloys processed by an extrusion process were collected, and Table 2 shows the statistical analysis of mechanical properties in the data samples. Figure 3 shows the correlation heat map of ultimate tensile strength (UTS), yield strength (YS), and elongation (EL) of the data samples of deformed magnesium alloys. The closer the value is to 1, the higher the correlation is; the correlation coefficient of YS and UTS is 0.79, and both of them have higher correlation.

Data preprocessing is required before model training to ensure the accuracy of the model prediction results. By normalizing and discretizing the training data, the interference of extreme and abnormal data in metallographic images is reduced. At the same time, the pictures are grayed out to make the model prediction more accurate and stable. The dataset of this model includes the training set, validation set, and test set. Z-score normalization is applied to the dataset to transform the data into a standard normal distribution with a mean of 0 and standard deviation of 1, as shown in Equation (2).

(2) $$x^{*}=\frac{x-\bar{x}}{\sigma }$$

where $\bar{x}$ is the mean of the dataset, and σ is the standard deviation of the dataset.

### 3.3. Construction of CNN Structure

In this study, a CNN model was used for the prediction of the room-temperature mechanical properties of the alloys, which was constructed using convolution plus pooling through a twice fully connected network architecture. In the use of the framework, this project adopts the PyTorch framework (https://pytorch.org/), the convolution kernel is 3 × 3, 4-layer convolution, the step size is 2, the activation function is selected as the Rectified Linear Unit (Relu) function, maximum pooling is used, and the overall framework is shown in Table 3.

**Figure 4:**
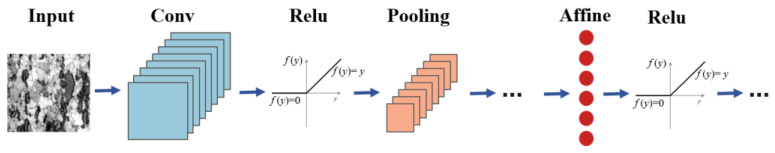
Convolutional neural network model structure.

The process of constructing the CNN model is shown in Figure 4, where firstly the first convolution operation layer is applied on the input, and nonlinear transformation is performed using the ReLU activation function; then, the output is subjected to the maximum pooling operation. In the second step, the second convolution layer is applied on the result of the first layer output, and transformations are performed using the ReLU function until the output of the fourth layer. The result of pooling the fourth layer is flattened (i.e., converted to one-dimensional), and the outputs of the first, second, and third fully connected layers are nonlinearly transformed using the ReLU activation function in turn.

The model training process is as follows: forward propagation to obtain the output prediction results of the model, calculate the loss value according to the prediction results and the actual mechanical properties, back-propagate the loss to obtain the gradient information of each parameter, and use the gradient information to update the parameters of the model. Finally, the loss values are accumulated to output the average loss value of the current training. The function uses the built-in optimizer of PyTorch for parameter updating and the cross-entropy loss function for loss calculation.

The process of model accuracy validation is as follows: load the validation set data in batches, obtain the output of the model through forward propagation, calculate the mode relative error, and, finally, output the absolute correctness on the training dataset. The mean square error loss function (MSELoss) is used for loss calculation in the regression task, and the construct is optimized using the stochastic gradient descent (SGD) algorithm for parameter optimization. The learning rate (Learning rate) is 0.0001 and the training and validation process is performed in a loop for 800 times, and the relative correctness is output.

**Figure 5:**
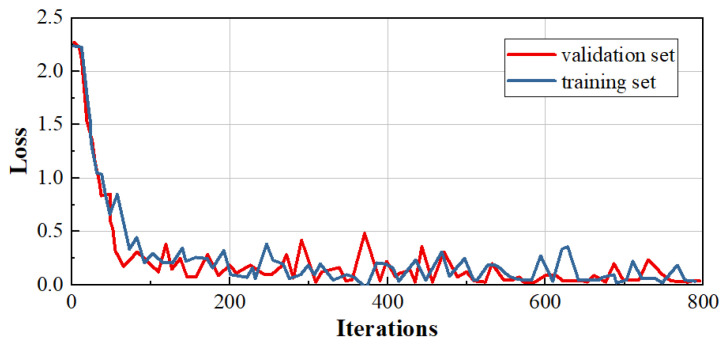
Error changes during model training.

Figure 5 shows the change in error during model training. The red color is the validation set, and the blue color is the training set. It can be seen that after 800 trainings, the loss is stable at around 0; that is, the model training error is very small.

### 3.4. Verification of CNN Model

**Figure 6:**
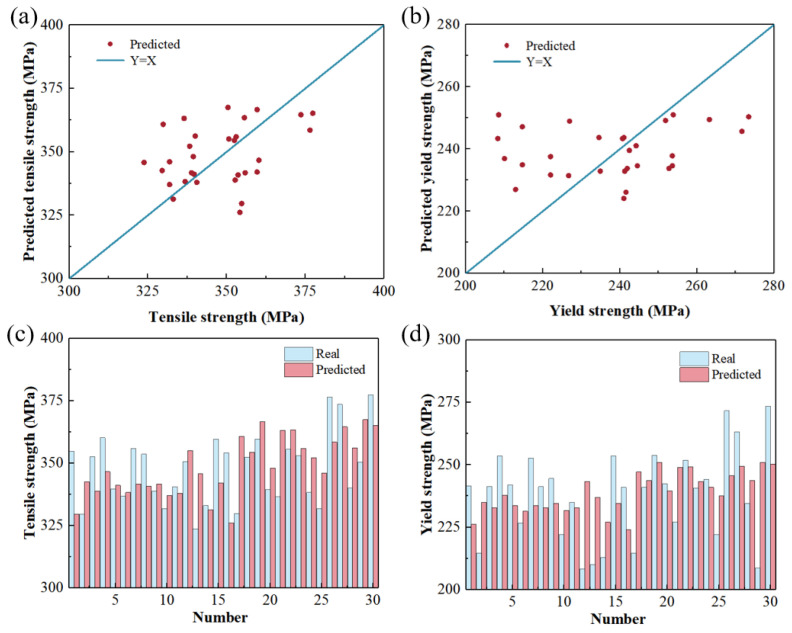
Comparison of the model’s predicted and true values before optimization. (**a**,**c**) Tensile strength; (**b**,**d**) yield strength.

The test data are input into the trained mechanical property prediction model. The model prediction results are shown in Figure 6. The horizontal axis in the image is the measured value, and the vertical axis is the model prediction value. The straight blue line Y = X in the Figure indicates that the predicted value is equal to the actual value, which is the ideal model prediction result straight line. The red scattered points are the predicted values. The closer these scattered points are to the straight line, Y = X, the better the prediction effect of the model. As can be seen from the figure, the predicted values of the ultimate tensile strength and yield strength are generally consistent with the experimental data.

**Figure 7:**
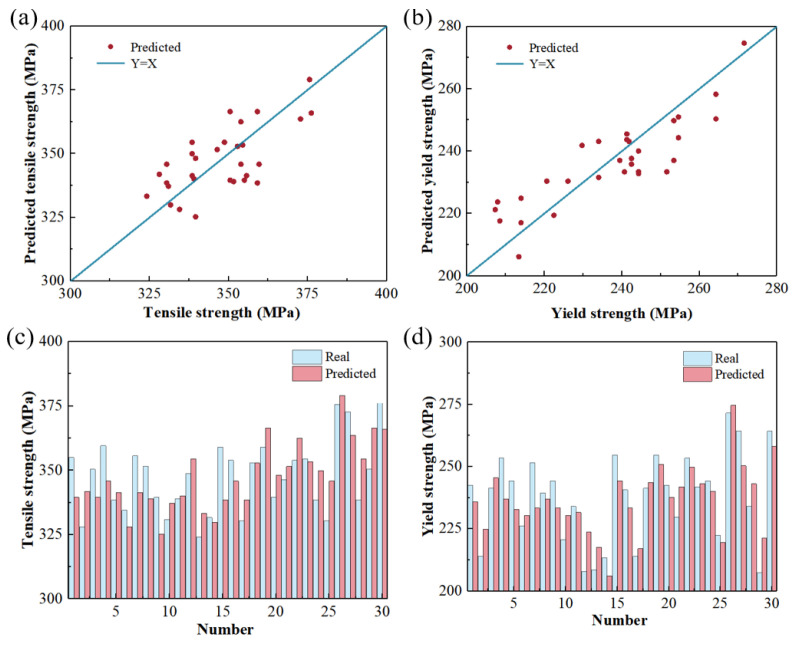
Comparison between the predicted true values after optimization. (**a**,**c**) Tensile strength; (**b**,**d**) yield strength.

**Figure 8:**
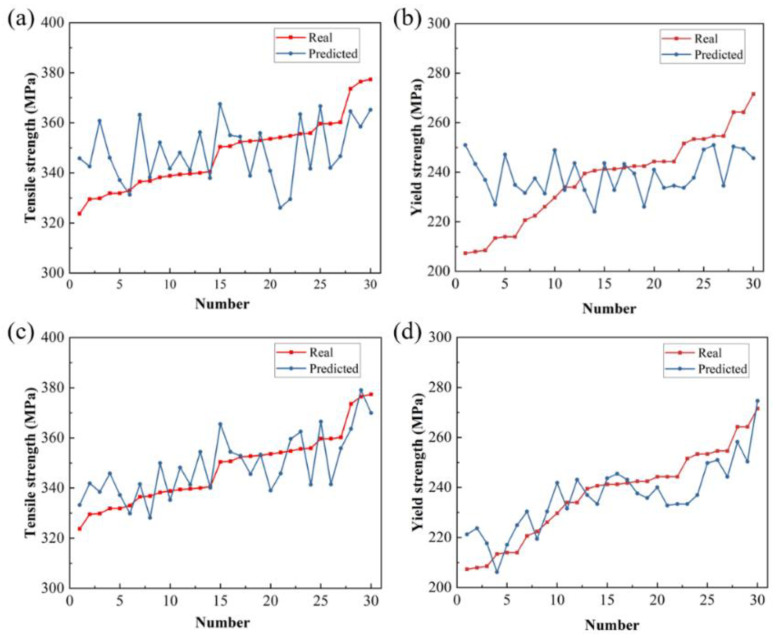
Comparison of predicted and true values before and after model optimization. (**a**,**b**) Tensile strength and yield strength before optimization; (**c**,**d**) Tensile strength and yield strength after optimization.

**Figure 9:**
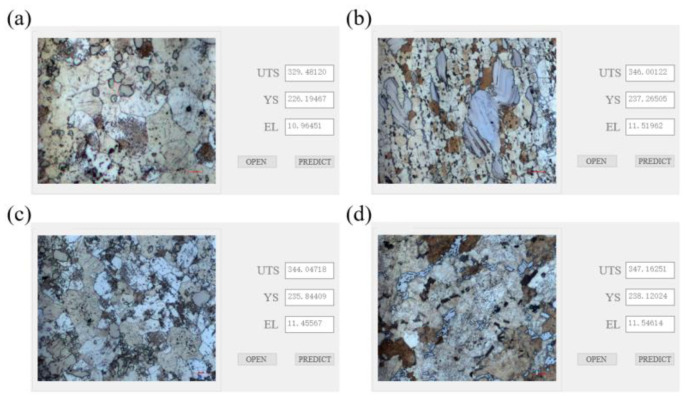
Prediction process of partial test data (**a**–**d** magnification of 500).

In order to improve the accuracy of the model, the model structure, parameters, etc., are optimized to reduce the generalization error caused by overfitting. Optimization is mainly carried out from the aspects of data enhancement, model regularization, terminating training in advance according to the model training situation, and shutting down some neurons. Then, the mechanical property prediction model is retrained through the convolutional neural network, and the training process before optimization is repeated to obtain the final model. The test set data are input into the model to obtain the prediction results. As shown in Figure 7, the red scattered points are the prediction results of the entire test set, which are evenly distributed near the blue Y = X straight line. After calculation, the mean square error between the predicted value and the actual value of the tensile strength is 3.25%, and the mean squared error (MSE) for tensile strength predictions compared to actual values is 3.25%, with an R^2^ coefficient of 0.43. For yield strength, the MSE is 4.77%, and the R^2^ coefficient is 0.74. Before optimization, the MSE for tensile strength and yield strength are 4.26% and 5.92%, with R^2^ coefficients of 0.11 and 0.04, respectively. The prediction accuracy of the optimized model is improved by 21.6%. Figure 8 contains a diagram of the degree of fit of the mechanical property prediction values before and after model optimization. It can be seen that the degree of fit between the predicted values of tensile strength and yield strength and the actual values has been greatly improved after optimization. As shown in Figure 9, the metallographic images of samples ①, ②, and ③ are input into the model to predict the mechanical properties. The results are shown in Table 4.

## 4. Results and Discussion

**Figure 10:**
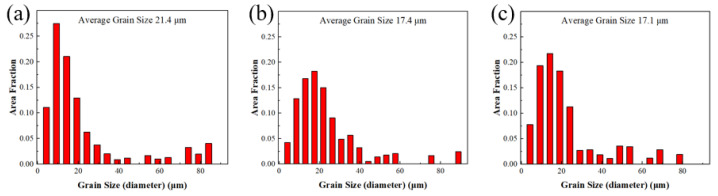
Average grain size distribution of samples deformed by different processes. (**a**) Sample ①; (**b**) sample ②; and (**c**) sample ③.

As shown in Table 4 above, the tensile strength of samples ①, ②, and ③ are 300 MPa, 336 MPa, and 357.8 MPa, respectively; the yield strength is 225 MPa, 237 MPa, and 245.6 MPa, respectively; and the elongation after fracture is 9.74%, 11.7%, and 10.15%, respectively. The tensile strength and yield strength of samples ①, ②, and ③ increase successively, which is significantly related to the grain size of the deformed rare-earth magnesium alloy. We used the electron backscatter diffraction (EBSD) analysis technique of a SU5000 thermal field-emission electron microscope system for grain size characterization and microanalysis of rare-earth magnesium alloy samples. As shown in Figure 10, the average grain sizes of samples ①, ②, and ③ are 54.2 μm, 15.29 μm, and 10.1 μm, respectively. It can be seen that the yield strength increases with the decrease in grain size, showing a negative correlation, which conforms to the Hall–Petch relationship, as shown in Formula (3).

(3) $$\sigma_{y}=\sigma_{0}+Kd^{-1/2}$$

where σ_y_ is the yield strength of the magnesium alloy after tensile fracture, σ_0_ is the friction resistance when a single dislocation moves in the magnesium alloy, K is the constant, and d is the average grain size of the magnesium alloy.

**Figure 11:**
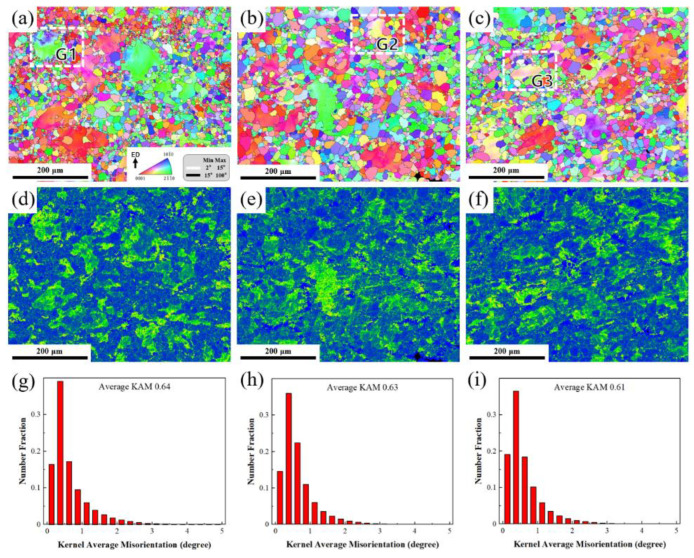
(**a**–**c**) Inverse pole figure maps of samples ①②③; (**d**–**f**) Kernel Average Misorientation maps of samples ①②③; and (**g**–**i**) frequency distribution of KAM values of samples ①②③.

As shown in Figure 11a–c, the microstructure of sample ① is mainly composed of coarse grains and a small amount of fine dynamic recrystallization (DRX) grains. Fine DRX grains are also observed inside some coarse grains. This may be due to the kinking behavior of the coarse grains. The fine DRX grains nucleate on the kink band and gradually grow. The alloy structure of sample ② is more uniformly distributed, with some bimodal microstructures, and fine spherical grains gather at the grain boundaries of large grains. The grains of the alloy structure of sample ③ are more refined and uniform, and the uniformity and regularity of the recrystallized grains are improved. Among them, the reasons for the higher mechanical properties of sample ③ are as follows: first, the fine RE-rich phase precipitated is dispersed, which hinders the movement of dislocations and thus strengthens the alloy strength; second, the special stacking structure of the long-period-stacking-ordered (LPSO) phase hinders the movement of dislocations; and third, the static recrystallization refines the grains and plays a role in grain refinement strengthening. The distribution of average orientation difference is consistent with the distribution of low-angle grain boundaries (LAGBs) in the anti-pole diagram due to density-related deformation accumulation. Figure 11d–i is the Kernel Average Misorientation (KAM) diagram of samples ①②③, which reflects the geometrically necessary dislocation density to a certain extent. When the theoretical shear strain is 0, the KAM value is low because the deformation at the center is small, and there is not enough stress to cause local dislocations to accumulate, so there is no increase in dislocation density. After upsetting–extrusion deformation, the dislocation density of the original coarse grains is concentrated, which provides sufficient nucleation energy for dynamic recrystallization.

**Figure 12:**
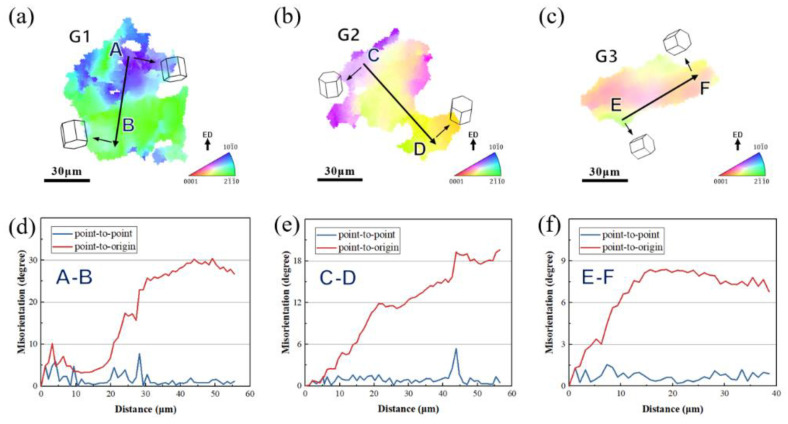
(**a**–**c**) Inverse pole figure maps of grains G1–G3 in Figure 11; (**d**–**f**) line profile of misorientation angle along the arrows AB in (**a**–**c**).

To further compare the intragranular distributions of inverse pole figure (IPFs) in those specimens after different process deformation, Figure 12 contains enlarged images of the grains in the G1–G3 region in Figure 11a–c, which, respectively, present the DRX behavior of the grains of samples ①②③. There is no color change inside a large number of grains in samples ①②③. In Figure 12a, grain G1 is composed of blue and green regions. The line graph along the black arrow, AB (Figure 12d), shows that the misalignment angle gradually increases to 30, indicating that a continuous change in direction has occurred in G1.

## 5. Conclusions

(1) A CNN was used to establish an artificial neural network model for predicting the mechanical properties of a Mg-9Gd-Y-Zn-Zr alloy. Compared with parameterizing microstructure features and ignoring some of them, this model achieves the prediction of mechanical properties of magnesium alloys by directly linking the mechanical properties of rare-earth magnesium alloys with metallographic diagrams through the convolutional neural network. The results show that the average relative errors of the prediction results of tensile strength and yield strength are 1.90% and 3.14%, respectively, which are less than the expected error of 5%.
(2) Three different processes were used to deform rare-earth magnesium alloy specimens, and the average error of the mechanical property prediction results was less than 5%. Therefore, the convolutional neural network model proposed in this paper can be used as a prediction model for the mechanical properties of a Mg-9Gd-Y-Zn-Zr alloy.
(3) After three-pass upsetting and annealing at 420 °C for 5 h, the mechanical properties of the Mg-9Gd-Y-Zn-Zr alloy were the best, which was attributed to grain refinement and dislocation strengthening.

## Figures and Tables

**Table 1:** Chemical composition of rare-earth magnesium alloy (wt.%).

Element	Gd	Y	Zn	Zr	Al	Ca	Si	Mg
Content (wt.%)	9.32	4.02	2.16	0.48	<0.01	<0.01	<0.01	others

**Table 2:** Statistical analysis of mechanical properties in the data samples.

Number	Mechanical Properties	Max	Min	Mean
1	UTS/MPa	379.1	306.8	348.96
2	YS/MPa	272.4	207.8	239.37
3	EL/%	12.5	8.0	11.63

**Table 3:** General framework of the CNN.

Layer	Output Shape	Param
Conv2d-1	[3, 20, 239, 239]	200
MaxPool2d-2	[3, 20, 120, 120]	0
Conv2d-3	[3, 30, 59, 59]	5430
MaxPool2d-4	[3, 20, 30, 30]	0
Conv2d-5	[3, 40, 28, 28]	10,840
MaxPool2d-6	[3, 40, 15, 15]	0
Conv2d-7	[3, 40, 13, 13]	14,440
MaxPool2d-8	[3, 40, 7, 7]	0
Linear-9	[3, 500]	980,500
Linear-10	[3, 100]	50,100
Linear-11	[3, 3]	303

**Table 4:** Part of comparison between the actual values and the predicted values of the model.

Number	UTS			YS			EL		
	Real	Predicted	Error	Real	Predicted	Error	Real	Predicted	Error
	/MPa	/MPa	%	/MPa	/MPa	%	%	%	%
1	300	310.6	3.53	225	213.6	5.07	9.74	10.3	5.75
2	336	334.5	0.45	237	229.7	3.08	11.7	11.14	4.79
3	357.8	373.36	4.35	245.6	255.58	4.06	10.15	10.45	2.96
4	350.1	341.61	2.43	244.1	234.35	3.99	11.5	11.36	1.22
5	345	339.41	1.62	236.1	232.8	1.40	11.8	11.3	4.24
6	361.2	364.67	0.96	245.7	249.81	1.67	11.9	12.16	2.18
7	345.6	342.27	0.96	242.4	234.83	3.12	12.4	11.89	4.11
8	331.8	338.07	1.89	221.6	231.9	4.65	11	11.27	2.45
9	332.7	329.66	0.91	223.7	226.35	1.18	11.2	10.96	2.14
Mean			1.90			3.14			3.32

## Data Availability

The original contributions presented in the study are included in the article, further inquiries can be directed to the corresponding author.
